# Functional disability, diabetes and hypertension among adults in the United States, August 2021–August 2023

**DOI:** 10.1016/j.pmedr.2026.103664

**Published:** 2026-10-09

**Authors:** Ying Liu, Piaopiao Chen, Yanmei Su, Juan Xie, Weiquan Zeng, Chun Pan

**Affiliations:** a Department of Traditional Chinese Medicine and Acupuncture, Loudi Central Hospital, Loudi, Hunan, China

**Keywords:** Functional disability, Diabetes, Hypertension, Population survey, Age standardization

## Abstract

**Objective:**

To describe diabetes and hypertension prevalence, diagnosis status and measured-threshold outcomes by functional disability.

**Methods:**

We analyzed the National Health and Nutrition Examination Survey conducted in the United States during August 2021–August 2023. Adults aged 18 years or older were classified using six Washington Group functioning domains. Outcome-specific weights and direct age standardization were used. Disability-minus-no-disability differences and 95% confidence intervals accounted for the complex survey design.

**Results:**

Among 8101 adults with complete functioning data, total diabetes and hypertension were classifiable in 5974 and 6172. Standardized total-disease differences were 9.3 percentage points (5.8, 12.7) for diabetes and 8.3 (3.1, 13.6) for hypertension. Undiagnosed-disease differences among eligible adults were 0.6 (−0.4, 1.7) and −3.4 (−7.2, 0.5), respectively. Among hypertension cases with known diagnosis status, the undiagnosed fraction was lower with disability: −14.1 (−19.9, −8.3). Conditional diabetes estimates were imprecise and sensitive to sparse younger-age cells.

**Conclusions:**

Adults with functional disability had higher standardized total diabetes and hypertension prevalence. Diagnosis-status and measured-threshold findings depended on the condition and denominator; they do not establish equivalent care or explain the origins of prevalence differences.

## Highlights

• Adults with disability had higher standardized diabetes and hypertension burdens.
• Undiagnosed disease estimates depended on the denominator and condition.
• Young diabetes subgroups limited the precision of conditional estimates.
• Cross-sectional differences do not establish equivalent detection or management.

## 1 Introduction

Functional disability describes difficulty in activities that can be shaped by health conditions and the surrounding environment (Verbrugge and Jette, 1994). The Washington Group Short Set on Functioning (WG-SS) provides a population measure across six domains (Madans et al., 2011). Recent interview data from the United States show a higher prevalence of diagnosed diabetes among adults with disability (Bardenheier et al., 2025). Interview diagnosis alone does not identify adults whose glycated hemoglobin (HbA1c) is above a diabetes threshold without a reported diagnosis.

Related evidence limits the contribution that a further survey analysis can claim. Shah et al. (2026) examined hypertension prevalence, awareness and control in the same National Health and Nutrition Examination Survey (NHANES) cycle using an expanded disability measure. Zhou et al. (2023) jointly examined diabetes and hypertension prevention among stroke survivors in earlier cycles. A contemporary general-adult analysis can add a six-domain description of HbA1c-based diabetes burden and diagnosis status, with hypertension providing a contextual comparison. Parallel presentation identifies condition-specific descriptive patterns; it does not test whether disability has a different effect on the two diseases.

We compared total disease prevalence, the population burden of undiagnosed disease, the fraction undiagnosed among disease cases, and measured-threshold outcomes among previously diagnosed or treated adults. Direct standardized differences quantify the uncertainty in these comparisons. Because classification and age composition influence these estimates, we also assessed finer age groups, an alternative blood pressure threshold, measurement availability and the handling of incomplete functioning responses. These are retrospective descriptive analyses; no prospectively registered hypothesis is claimed.

## 2 Methods

### 2.1 Study design and population

NHANES August 2021–August 2023 sampled the civilian, noninstitutionalized population of the United States using a stratified, multistage probability design (Terry et al., 2024; National Center for Health Statistics, 2024a). We linked public interview, functioning, diabetes, blood pressure and glycohemoglobin files by participant identifier. Adults aged 18 years or older with valid responses to all six functioning domains formed the interview cohort. Each disease cohort then required a positive corresponding component weight and a classifiable outcome. This retained the maximum classifiable sample conditional on complete functioning data and the applicable weight; measured-threshold analyses additionally required the relevant measurement.

### 2.2 Measures

Functional disability was a lot of difficulty or inability in at least one of seeing, hearing, walking or climbing steps, remembering or concentrating, communicating, and self-care (Hanass-Hancock et al., 2023). Responses were coded from FNQ410, FNQ430, FNQ440, FNQ460, FNQ450 and FNQ470, respectively. Refused, unknown or missing responses were not negative responses. Requiring six valid answers is stricter than the published FNDADI indicator, which can classify some partial responders. The published indicator was evaluated separately as a sensitivity analysis. Exact wording, response codes and denominator rules appear in Supplementary Table S1.

Diagnosed diabetes was an affirmative response to DIQ010, which asks whether a doctor or other health professional ever told the respondent that they had diabetes. No and borderline responses were coded as no established diagnosis. The question excludes pregnancy-only diagnosis in its wording for women aged 20 years or older; that qualifier is not administered uniformly to all female adults. Total diabetes was diagnosed diabetes or HbA1c ≥ 6.5% (American Diabetes Association Professional Practice Committee, 2024a). Undiagnosed diabetes required HbA1c ≥ 6.5% and a known absence of established diagnosis. Its numerator was expressed over all eligible adults and, separately, over total diabetes cases with known diagnosis status. HbA1c ≥ 7.0% was evaluated among diagnosed adults with an available measurement. This surveillance threshold does not assess individualized treatment targets (American Diabetes Association Professional Practice Committee, 2024b).

Total hypertension was an ever-told diagnosis (BPQ020), current prescribed antihypertensive medication (BPQ150), or mean systolic pressure ≥ 130 or diastolic pressure ≥ 80 mmHg (Whelton et al., 2018). Means used up to three available oscillometric readings; systolic and diastolic availability was paired in the released data. BPQ150 is asked only after an affirmative BPQ020 response. No adults reported current medication while reporting no or unknown prior diagnosis. A routed medication skip after a negative diagnosis response was distinguished from unknown diagnosis. Undiagnosed hypertension required a positive measured threshold, no prior diagnosis and no current treatment. It was expressed over eligible adults and over hypertension cases with known diagnosis status. The measured-threshold outcome used diagnosed or treated adults with blood pressure measurements as its denominator. Pregnancy was not an exclusion criterion.

Known diagnosed adults remained classifiable for total disease, and as not undiagnosed, when measurement was missing, provided their component weight was positive. A positive measurement could also establish total disease with unknown history, but could not establish whether it was undiagnosed. Refusal or unknown history was never coded as absence of diagnosis. Education and family income-to-poverty ratio described the cohorts; neither was an exclusion criterion. Education was asked only from age 20, and not-asked responses were separated from other missingness. Supplementary Table S2 details sample selection and missingness.

### 2.3 Statistical analysis

Survey designs incorporated masked strata and primary sampling units before domain restriction. Functioning-only estimates used interview weights (WTINT2YR); analyses combining functioning with HbA1c or diabetes used phlebotomy weights (WTPH2YR), and hypertension analyses used examination weights (WTMEC2YR), including cohort characteristics and functioning prevalence (National Center for Health Statistics, 2024a, National Center for Health Statistics, 2024b). Component weights account for interview-to-examination or blood-collection nonresponse. Residual exclusions and inclusion profiles were unweighted sample descriptions (Supplementary Tables S2 and S6). No additional response model or imputation was fitted.

Disease estimates were directly standardized to the projected 2000 United States population restricted to adulthood (Klein and Schoenborn, 2001). Exact single-age counts from the National Cancer Institute (n.d.) produced weights of 53.1%, 29.9% and 17.0% for ages 18–44, 45–64 and 65 years or older; calculations used unrounded weights. These broad categories balance age adjustment and sample information, but cannot remove age differences within categories. Raw weighted estimates and age-cell counts were retained for interpreting the conditional outcomes.

Age-by-disability proportions were estimated jointly. Fixed standard-population weights were applied to the full Taylor-linearized covariance matrix, including cross-age and cross-group covariance. Disability-minus-no-disability differences and 95% confidence intervals (CIs) used the resulting contrast variance and a t distribution with 15 design degrees of freedom. Group-CI overlap was not used to judge differences (Schenker and Gentleman, 2001). Unstandardized functioning and demographic prevalence intervals used a logit transformation with Taylor standard errors. We reported absolute differences without redundant *p*-values and made no formal between-disease comparison.

Sensitivity analyses used seven age groups (18–24, 25–34, 35–44, 45–54, 55–64, 65–74 and 75 years or older), a hypertension threshold of ≥140 systolic or ≥90 diastolic mmHg, measurement-complete denominators, and the published disability indicator. Empty age-cell denominators were reported as not estimable; small denominators and sparse events were flagged for caution, not presented as formal reliability certification. Unweighted included/excluded demographic profiles characterized the observed sample exclusions. These checks address the classification and precision concerns described above, not all possible missing-data mechanisms. Analyses used R version 4.5.1 and the survey package (Lumley, 2004).

### 2.4 Ethics

NHANES protocols and procedures were approved by the National Center for Health Statistics Research Ethics Review Board. Verbal consent preceded household questionnaires, and written informed consent preceded examination (Terry et al., 2024). This secondary analysis used only publicly available, de-identified data and involved no participant contact. It met the data curator (NCHS) guidelines for the protection of human subjects concerning safety and privacy and was exempt from institutional ethics review. The statement of exemption is retained on file with the authors' institution.

## 3 Results

### 3.1 Analytic samples and functioning

Of 8153 interviewed adults, 52 lacked complete functioning responses, leaving 8101: 6569 without disability and 1532 with disability. Weighted disability prevalence was 16.4% (95% CI, 14.5, 18.6). Functioning prevalence and cohort composition are reported using the corresponding cohort weights (Table 1, Table 2). Income-to-poverty ratio was missing for 1369 adults (16.9% unweighted); 340 aged 18–19 were not asked the adult education question.

**Table 1:** Prevalence of functional disability across analytic cohorts of adults in the United States, August 2021–August 2023.

Domain	Interview cohort 8101 adults	Diabetes cohort 5974 adults	Hypertension cohort 6172 adults
Any of the six domains	16.4 (14.5, 18.6)	16.6 (14.1, 19.4)	16.8 (14.4, 19.6)
Seeing	3.7 (3.1, 4.4)	3.4 (2.6, 4.5)	3.5 (2.8, 4.6)
Hearing	2.6 (2.2, 3.0)	2.6 (2.2, 3.1)	2.6 (2.2, 3.1)
Walking or climbing steps	7.3 (6.4, 8.4)	7.0 (6.0, 8.2)	7.2 (6.1, 8.4)
Remembering or concentrating	6.5 (5.6, 7.5)	6.7 (5.7, 7.9)	6.8 (5.8, 8.0)
Communicating	1.5 (1.2, 1.9)	1.3 (0.9, 1.8)	1.4 (1.0, 1.9)
Self-care	1.2 (1.0, 1.5)	0.8 (0.6, 1.2)	0.9 (0.6, 1.2)

Values are weighted percentages (95% confidence intervals); cohort sizes are unweighted. Functional disability is a lot of difficulty or inability in at least one domain. All rows require complete responses to the same six domains.

Interview, diabetes and hypertension columns use interview, phlebotomy and examination weights, respectively. Diabetes and hypertension cohorts contain all classifiable total-disease outcomes with positive corresponding weights. Domain percentages overlap.

**Table 2:** Demographic composition of analytic cohorts of adults in the United States, August 2021–August 2023.

Characteristic	Interview cohort 8101 adults	Diabetes cohort 5974 adults	Hypertension cohort 6172 adults
Age, years			
18–29	1239 (20.0)	830 (20.0)	877 (19.6)
30–44	1751 (26.6)	1279 (26.4)	1299 (26.1)
45–64	2546 (31.7)	1970 (32.0)	2022 (32.3)
65 or older	2565 (21.7)	1895 (21.6)	1974 (22.0)

Sex			
Male	3632 (48.5)	2692 (48.5)	2790 (48.7)
Female	4469 (51.5)	3282 (51.5)	3382 (51.3)

Race and ethnicity			
Mexican American	596 (7.7)	428 (7.3)	434 (7.2)
Other Hispanic	813 (9.3)	619 (9.7)	628 (9.5)
Non-Hispanic White	4674 (59.6)	3503 (59.8)	3606 (60.1)
Non-Hispanic Black	1039 (11.1)	710 (11.0)	758 (10.9)
Non-Hispanic Asian	440 (6.0)	329 (6.2)	344 (6.1)
Other or multiracial	539 (6.3)	385 (6.1)	402 (6.1)

Education			
Below ninth grade	372 (3.4)	273 (3.7)	286 (3.7)
Ninth to eleventh grade	660 (6.4)	442 (6.0)	466 (6.1)
High school or equivalent	1738 (25.5)	1201 (25.5)	1256 (25.3)
Some college or associate degree	2355 (28.5)	1751 (28.5)	1793 (28.3)
College graduate or above	2619 (32.9)	2074 (33.0)	2110 (33.2)
Not asked at ages 18–19	340 (3.2)	231 (3.3)	257 (3.3)
Missing at age 20 or older	17 (0.2)	2 (0.0)	4 (0.0)

Family income-to-poverty ratio			
<1.30	1574 (16.3)	1156 (17.4)	1214 (17.3)
1.30 to below 3.50	2532 (30.6)	1962 (32.6)	2026 (32.6)
3.50 or higher	2626 (35.3)	2091 (36.4)	2130 (36.3)
Missing	1369 (17.9)	765 (13.6)	802 (13.8)

Functional disability			
No disability	6569 (83.6)	4862 (83.4)	4999 (83.2)
Disability	1532 (16.4)	1112 (16.6)	1173 (16.8)

Values are unweighted counts (weighted percentages) within each entire cohort. Interview, phlebotomy and examination weights are used in the corresponding columns.

Education was asked at age 20 years or older. Not-asked and missing responses remain separate categories. Missing education or income does not exclude adults from disease analyses. Percentages may not sum to 100.0 because of rounding.

Among complete functioning respondents, 5999 had positive phlebotomy weights and 5972 had HbA1c measurements. Total diabetes was classifiable in 5974, including three diagnosed adults without HbA1c; one measured adult lacked classifiable diabetes status. Of 6306 with positive examination weights, 6093 had blood pressure measurements. Total hypertension was classifiable in 6172, including 81 known cases without blood pressure and two measured-positive adults with unknown prior diagnosis. These two were excluded from the undiagnosed-status analyses, whose adult denominator was 6170. The total hypertension case count was 3470; 3468 had known diagnosis status (Supplementary Tables S2 and S3).

Incomplete functioning or unclassifiable total disease excluded 55 of 6029 adults with positive phlebotomy weights (0.9%) and 165 of 6337 with positive examination weights (2.6%). Supplementary Tables S2 and S6 provide unweighted measurement-availability counts and sample proportions by disability status, missingness, and included/excluded demographic profiles. These sample descriptions do not estimate national HbA1c or blood pressure distributions or establish absence of selection bias.

### 3.2 Disease prevalence and conditional outcomes

Age-standardized total diabetes prevalence was 19.8% with disability and 10.5% without disability, a difference of 9.3 percentage points (95% CI, 5.8, 12.7). Total hypertension prevalence was 54.2% and 45.9%, respectively: difference 8.3 points (3.1, 13.6). Table 3 provides group-specific intervals and every unweighted numerator and denominator; Fig. 1 displays the direct contrasts.

**Table 3:** Age-standardized diabetes and hypertension estimates and disability-group differences among adults in the United States, August 2021–August 2023.

Outcome and denominator population	Without disability	With disability	Difference in percentage points
Total diabetes	637/4862 10.5 (9.4, 11.6)	337/1112 19.8 (16.3, 23.2)	9.3 (5.8, 12.7)
Undiagnosed diabetes among all eligible adults	111/4862 1.9 (1.4, 2.5)	41/1112 2.6 (1.7, 3.4)	0.6 (−0.4, 1.7)
Undiagnosed diabetes among diabetes cases†	111/637 21.1 (15.6, 26.6)	41/337 17.2 (5.9, 28.4)	−3.9 (−16.4, 8.5)
Glycated hemoglobin ≥7.0% among diagnosed adults†	265/523 57.6 (50.5, 64.7)	156/296 65.1 (50.3, 80.0)	7.5 (−7.5, 22.6)
Total hypertension	2644/4999 45.9 (43.6, 48.2)	826/1173 54.2 (49.8, 58.7)	8.3 (3.1, 13.6)
Undiagnosed hypertension among all eligible adults	981/4997 19.7 (17.9, 21.6)	173/1173 16.4 (13.5, 19.3)	−3.4 (−7.2, 0.5)
Undiagnosed hypertension among cases with known diagnosis status	981/2642 49.3 (45.9, 52.6)	173/826 35.2 (30.6, 39.8)	−14.1 (−19.9, −8.3)
Blood pressure ≥ 130/80 mmHg among diagnosed or treated adults	980/1620 61.7 (57.1, 66.3)	321/613 48.6 (40.4, 56.7)	−13.1 (−21.1, −5.2)

Group cells show unweighted events/eligible adults, then standardized percentage (95% confidence interval). Differences are disability minus no disability. Standardization uses projected 2000 United States ages 18–44, 45–64 and ≥65. Phlebotomy and examination weights apply to diabetes and hypertension, respectively; intervals use joint Taylor covariance and 15 design degrees of freedom.

Total diabetes is diagnosis or glycated hemoglobin ≥6.5%; total hypertension is diagnosis, treatment or systolic pressure ≥ 130 or diastolic pressure ≥ 80 mmHg. Undiagnosed disease requires measured elevation and known absence of diagnosis (and hypertension treatment). Known diagnoses remain classifiable without measurement; two unknown-history hypertensive adults enter only total-disease estimates. Complete definitions are in Supplementary Table S1.

†Young disability cells contain 20 diabetes cases (five undiagnosed) and 15 diagnosed adults with glycated hemoglobin (11 ≥ 7.0%); they carry 53.1% of the standard weight. Crude estimates are in Supplementary Table S3. These imprecise estimates do not establish equivalence; clinical targets are individualized.

**Fig. 1:**
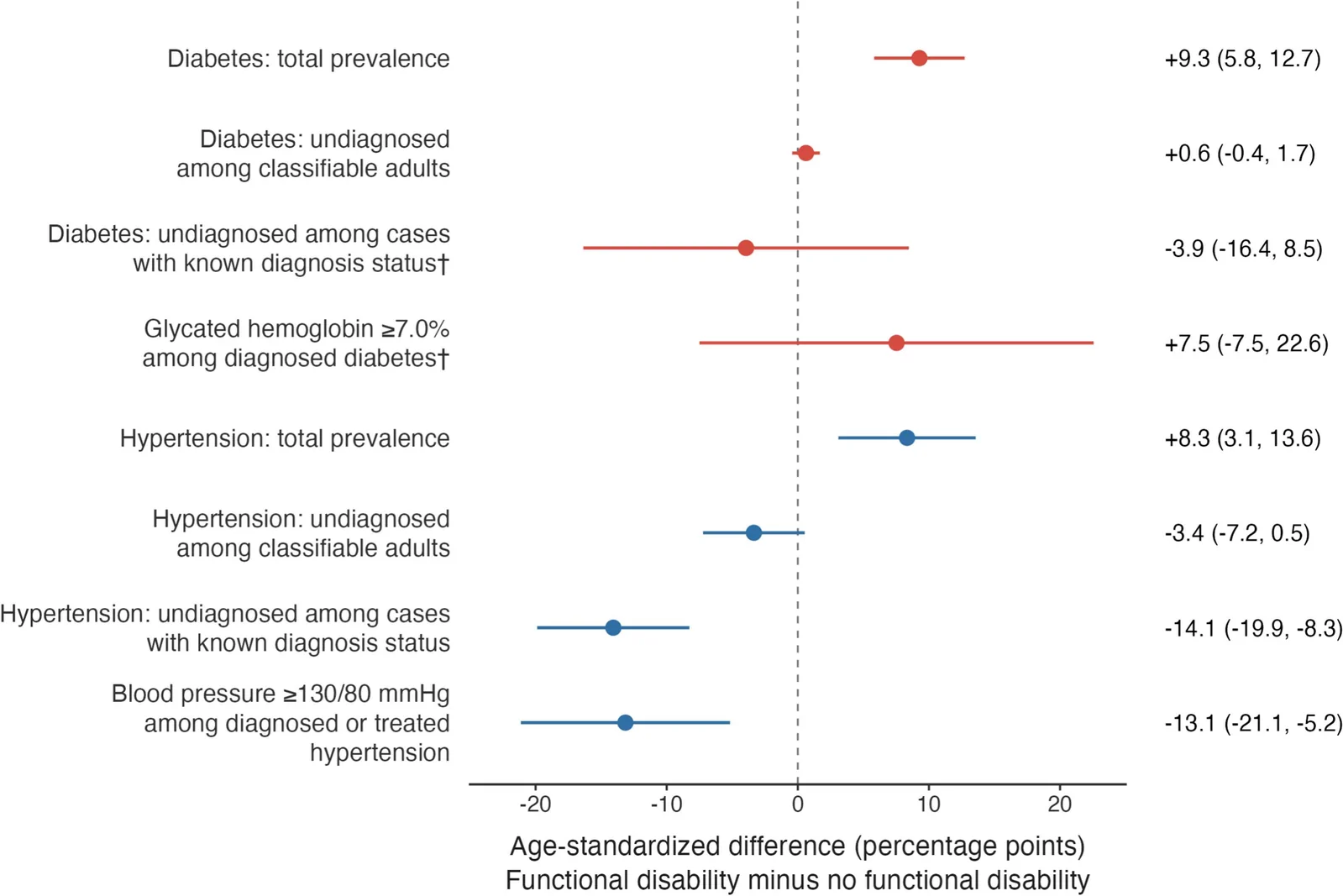
Age-standardized differences in diabetes and hypertension outcomes by functional disability among adults in the United States, August 2021–August 2023. Points show disability-minus-no-disability differences in percentage points; bars show 95% confidence intervals using joint survey covariance and 15 design degrees of freedom. Red indicates diabetes and blue hypertension. The vertical line marks zero difference. Eligible populations and counts are provided in Table 3. Standardization uses ages 18–44, 45–64 and 65 years or older. †Conditional diabetes estimates are unstable: the younger disability cells contain 20 diabetes cases and 15 diagnosed adults with glycated hemoglobin, and carry 53.1% of the standard weight. Intervals including zero do not establish equivalence. The glycated hemoglobin threshold does not assess individualized treatment targets.

Undiagnosed diabetes among eligible adults differed by 0.6 points (95% CI, −0.4, 1.7); undiagnosed hypertension differed by −3.4 points (−7.2, 0.5). Both intervals included zero and allowed differences in either direction. Among disease cases with known diagnosis status, undiagnosed diabetes differed by −3.9 points (−16.4, 8.5), whereas undiagnosed hypertension differed by −14.1 points (−19.9, −8.3). These are conditional case fractions, distinct from population prevalence.

Among diagnosed adults with HbA1c, the ≥7.0% proportion differed by 7.5 points (95% CI, −7.5, 22.6). Among diagnosed or treated adults with measured blood pressure, the ≥130/80 mmHg proportion differed by −13.1 points (−21.1, −5.2). The conditional diabetes estimates were unstable: the disability group aged 18–44 contained 20 diabetes cases, including five undiagnosed, and 15 diagnosed adults with HbA1c, including 11 at or above 7.0%. These cells receive 53.1% of the standard population weight. Crude companion estimates and cell counts are provided in Supplementary Table S3.

### 3.3 Sensitivity analyses

With seven age groups, total diabetes and hypertension differences were 9.9 points (95% CI, 6.3, 13.5) and 9.5 points (4.2, 14.9). Both conditional diabetes standardizations were not estimable because no disabled adults aged 18–24 entered their denominators. Other fine-age cells had sparse or zero events, limiting reassurance from these checks. Alternative blood pressure threshold results appear in Supplementary Table S4. Measurement-complete total-disease differences were 9.3 points for diabetes and 8.0 for hypertension. Using the published disability indicator retained 42 partial responders and yielded differences of 9.1 and 8.5 points, respectively (Supplementary Table S5).

## 4 Discussion

Adults with functional disability had higher age-standardized total diabetes and hypertension prevalence. Findings for diagnosis status and measured thresholds depended on the disease and denominator. The population undiagnosed-disease intervals did not exclude differences in either direction, and the diabetes case-fraction and HbA1c-threshold estimates were particularly imprecise. These results cannot establish that detection or management is equivalent between disability groups.

The diabetes analysis extends recent interview-based diagnosed-diabetes evidence by incorporating HbA1c and explicitly separating population burden from diagnosis status among cases (Bardenheier et al., 2025). It does not establish the effectiveness of a screening strategy. Joint diabetes and hypertension assessments already exist in stroke survivors (Zhou et al., 2023). Our general-adult, current-cycle comparison provides a narrower descriptive update. The hypertension findings contextualize that update and should be read as a form of descriptive replication.

Comparison with Shah et al. (2026) requires attention to estimands. Their same-cycle analysis used eight disability domains and nonpregnant adults; hypertension was measured elevation or current medication, without diagnosis history alone. Awareness and control used all hypertension cases as denominators. Here, the six-domain classification, inclusion of pregnancy, history-inclusive total hypertension and restriction of the threshold outcome to diagnosed or treated adults produce different populations and quantities. Agreement in direction does not make these estimates interchangeable.

Neither a lower undiagnosed case fraction nor a lower threshold-above proportion proves better care. Diagnosis, disease duration, treatment and survival can shape who enters a cross-sectional conditional denominator. The data do not separate contributions of disease occurrence, detection and management, or establish whether disability preceded disease. Reports of access difficulties among younger adults with disability and physicians' attitudes supply relevant context (Verlenden et al., 2022; Iezzoni et al., 2021), but those pathways were not measured here. Accessible prevention and treatment remain public health priorities; this study did not evaluate an intervention or identify its effects.

The analysis combines standardized functioning questions with laboratory and examination measurements and reports design-consistent uncertainty for the comparisons of interest. Several limits remain. Age standardization is descriptive and does not control socioeconomic, clinical or behavioral differences; broad groups leave residual age differences. Sparse young diabetes cells materially affect the conditional standardizations, while finer grouping can make them non-estimable. The wide intervals must remain part of the interpretation rather than being converted into reassuring claims.

One HbA1c measurement and one examination visit do not provide the repeated confirmation generally required for clinical diagnosis. HbA1c-only classification misses some diabetes identified by fasting or post-load glucose (American Diabetes Association Professional Practice Committee, 2024a). A fixed HbA1c threshold cannot capture appropriate individual targets, which may differ with functional or cognitive impairment (American Diabetes Association Professional Practice Committee, 2024b). Ever-told diagnosis is self-reported and does not establish disease duration or current clinical status. The functioning measure does not cover all disability experiences.

Finally, official weights address component participation but cannot guarantee correction of every nonresponse mechanism (National Center for Health Statistics, 2024a). Residual complete-case exclusions were limited within positive-weight cohorts, yet their small proportion and the measurement-complete sensitivity do not prove absence of selection bias. The published disability indicator classifies some partial negative responses without observing every domain, so its agreement is not validation of missingness assumptions. Institutionalized adults were outside the survey frame, and adults with more severe disability may be underrepresented.

## 5 Conclusions

Functional disability was associated descriptively with higher age-standardized total diabetes and hypertension prevalence among adults in the United States. Diagnosis-status and measured-threshold patterns were condition-specific and sensitive to their denominators and precision. They do not establish equivalent detection or management, temporal pathways, or effects of preventive interventions.

## CRediT authorship contribution statement

**Ying Liu:** Writing – original draft, Visualization, Methodology, Formal analysis, Data curation, Conceptualization. **Piaopiao Chen:** Writing – review & editing, Validation, Formal analysis, Data curation. **Yanmei Su:** Writing – review & editing, Validation, Formal analysis, Data curation. **Juan Xie:** Writing – review & editing, Validation. **Weiquan Zeng:** Writing – review & editing, Validation. **Chun Pan:** Writing – review & editing, Supervision, Methodology, Funding acquisition, Conceptualization.

## Ethics declaration

### Informed consent and patient details

Written informed consent to take part in the study and to publish the article has been obtained from all participants or their legal representatives. The privacy rights of participants have been observed.

### Studies in human

This study was performed in compliance with relevant laws, regulatory frameworks and guidelines where the research took place. This study was approved by the National Center for Health Statistics Research Ethics Review Board (Approval No. 2021-05).

## Funding

This work was supported by the 10.13039/501100004735Natural Science Foundation of Hunan Province (grant number 2025JJ70333 to Chun Pan). The funder had no role in study design, data collection and analysis, decision to publish, or preparation of the manuscript.

## Declaration of competing interest

The authors declare that they have no known competing financial interests or personal relationships that could have appeared to influence the work reported in this paper.

## Data Availability

Public data and codebooks are available from the National Health and Nutrition Examination Survey at https://wwwn.cdc.gov/nchs/nhanes/ for August 2021–August 2023. No new participant data were generated.

## Appendix A Supplementary data

Supplementary material 1 Supplementary Tables S1–S6. Variable definitions, sample flow and missingness, outcome estimates and age-cell precision, sensitivity analyses, participant profiles, and disability prevalence.

Supplementary material 2 Supplementary Tables S2 and S6. Participant flow and missing data, participant profiles, and subgroup and domain-specific disability prevalence.
