# Supplementary material for: Functional disability, diabetes and hypertension among adults in the United States, August 2021–August 2023
Source: Prev Med Rep. 2026 Oct 9;71:103664. doi: 10.1016/j.pmedr.2026.103664 (PMC13677198; doi:10.1016/j.pmedr.2026.103664)
Supplement: Supplementary file 1 [file mmc2.pdf]

Table S2. Participant flow and missing data

All counts and availability proportions are unweighted sample descriptions. Availability denominators are interview respondents with six valid functioning domains. These are not national HbA1c or blood pressure estimates. Flow rows overlap and are not sequential independent exclusions.

Sample flow

Step	Overall	No disability	Disability
Adult interview participants	8,153	6,569	1,532
Complete six Washington Group domains	8,101	6,569	1,532
Positive examination weight	6,306	5,111	1,195
Positive phlebotomy weight	5,999	4,879	1,120
glycated hemoglobin measurement available	5,972	4,860	1,112
Diabetes diagnosis response valid	8,099	6,567	1,532
Diabetes complete measured denominator	5,971	4,859	1,112
Diabetes maximal classifiable denominator	5,974	4,862	1,112
Known diabetes with missing glycated hemoglobin retained, positive phlebotomy weight	3	3	0
Blood pressure measurement available	6,093	4,959	1,134
Hypertension diagnosis/treated status valid	8,090	6,561	1,529
Hypertension complete measured denominator	6,089	4,956	1,133
Hypertension total classifiable with measured blood pressure	6,091	4,958	1,133
Hypertension maximal classifiable denominator	6,172	4,999	1,173
Known hypertension with missing blood pressure retained, positive examination weight	81	41	40

Disability groups require complete six-domain responses. The overall initial interview count also includes participants lacking a complete disability classification.

Variable response patterns

Population	Variable	N	Valid	Blank	Refused	Unknown	Structural blank	Unclear route blank	Not valid (%)
All adults	FNQ410	8,153	8,136	11	3	3	0	0	0.2
All adults	FNQ430	8,153	8,117	10	2	24	0	0	0.4
All adults	FNQ440	8,153	8,138	11	1	3	0	0	0.2
All adults	FNQ450	8,153	8,140	11	0	2	0	0	0.2
All adults	FNQ460	8,153	8,137	11	3	2	0	0	0.2
All adults	FNQ470	8,153	8,138	12	2	1	0	0	0.2
All adults	DIQ010	8,153	8,148	3	0	2	0	0	0.1
All adults	BPQ020	8,153	8,139	3	1	10	0	0	0.2
All adults	BPQ150	8,153	2,952	5,197	0	4	5,183	14	63.8
All adults	LBXGH	8,153	6,002	2,151			0	0	26.4
All adults	WTPH2YR	8,153	6,337	1,816			0	0	22.3
All adults	WTMEC2YR	8,153	8,153	0			0	0	0.0
All adults	DMDEDUC2	8,153	7,783	359	0	11	344	0	4.5
All adults	INDFMPIR	8,153	6,767	1,386			0	0	17.0
All adults	BPXOSY1	8,153	6,123	2,030			0	0	24.9
All adults	BPXOSY2	8,153	6,116	2,037			0	0	25.0

Variable response patterns

Population	Variable	N	Valid	Blank	Refused	Unknown	Structural blank	Unclear route blank	Not valid (%)
All adults	BPXOSY3	8,153	6,101	2,052			0	0	25.2
All adults	BPXODI1	8,153	6,123	2,030			0	0	24.9
All adults	BPXODI2	8,153	6,116	2,037			0	0	25.0
All adults	BPXODI3	8,153	6,101	2,052			0	0	25.2
Six valid domains	FNQ410	8,101	8,101	0	0	0	0	0	0.0
Six valid domains	FNQ430	8,101	8,101	0	0	0	0	0	0.0
Six valid domains	FNQ440	8,101	8,101	0	0	0	0	0	0.0
Six valid domains	FNQ450	8,101	8,101	0	0	0	0	0	0.0
Six valid domains	FNQ460	8,101	8,101	0	0	0	0	0	0.0
Six valid domains	FNQ470	8,101	8,101	0	0	0	0	0	0.0
Six valid domains	DIQ010	8,101	8,099	0	0	2	0	0	0.0
Six valid domains	BPQ020	8,101	8,090	0	1	10	0	0	0.1
Six valid domains	BPQ150	8,101	2,937	5,160	0	4	5,149	11	63.7
Six valid domains	LBXGH	8,101	5,972	2,129			0	0	26.3
Six valid domains	WTPH2YR	8,101	6,306	1,795			0	0	22.2
Six valid domains	WTMEC2YR	8,101	8,101	0			0	0	0.0
Six valid domains	DMDEDUC2	8,101	7,744	346	0	11	340	0	4.4
Six valid domains	INDFMPIR	8,101	6,732	1,369			0	0	16.9

Variable response patterns

Population	Variable	N	Valid	Blank	Refused	Unknown	Structural blank	Unclear route blank	Not valid (%)
Six valid domains	BPXOSY1	8,101	6,093	2,008			0	0	24.8
Six valid domains	BPXOSY2	8,101	6,086	2,015			0	0	24.9
Six valid domains	BPXOSY3	8,101	6,071	2,030			0	0	25.1
Six valid domains	BPXODI1	8,101	6,093	2,008			0	0	24.8
Six valid domains	BPXODI2	8,101	6,086	2,015			0	0	24.9
Six valid domains	BPXODI3	8,101	6,071	2,030			0	0	25.1

Structural and unclear-route blanks are components of the blank count. Not-valid percentages can include structural nonresponse and are not synonymous with avoidable item missingness. A blank entry for refusal/unknown means the concept is not applicable to that measurement.

Measurement and endpoint availability

Measure	Disability group	Sample N	Available	Available in sample (%)	Unavailable	Unavailable in sample (%)
Glycated hemoglobin measured	No disability	6,569	4,860	74.0	1,709	26.0
Glycated hemoglobin measured	Disability	1,532	1,112	72.6	420	27.4
Blood pressure measured	No disability	6,569	4,959	75.5	1,610	24.5
Blood pressure measured	Disability	1,532	1,134	74.0	398	26.0
Total diabetes classifiable	No disability	6,569	4,862	74.0	1,707	26.0
Total diabetes classifiable	Disability	1,532	1,112	72.6	420	27.4
Total hypertension classifiable	No disability	6,569	4,999	76.1	1,570	23.9

Measurement and endpoint availability

Measure	Disability group	Sample N	Available	Available in sample (%)	Unavailable	Unavailable in sample (%)
Total hypertension classifiable	Disability	1,532	1,173	76.6	359	23.4

Residual item exclusions after component participation

Component weight	Adults with positive weight	Incomplete six domains	Six valid domains	Missing measure	Total-disease unclassifiable	Combined excluded	Combined excluded (%)
Phlebotomy	6,029	30	5,999	27	25	55	0.9
Examination	6,337	31	6,306	213	134	165	2.6

Official component weights already address participation nonresponse. These counts do not establish that remaining values are missing at random. No additional imputation or inverse-probability weighting was performed.

Table S6. Participant profiles and subgroup disability prevalence

Included/excluded profiles show unweighted counts and sample proportions among interview respondents with six valid functioning domains. Subgroup disability prevalence uses interview weights. Domain prevalence uses the weight stated for each cohort.

Diabetes analysis inclusion profiles

Profile denominators: 2,127 excluded and 5,974 included. Category percentages use the corresponding profile denominator.

Characteristic	Category	Excluded count	Excluded (%)	Included count	Included (%)
Age (years)	18-29	409	19.2	830	13.9
Age (years)	30-44	472	22.2	1,279	21.4
Age (years)	45-64	576	27.1	1,970	33.0
Age (years)	65 and older	670	31.5	1,895	31.7
Sex	Male	940	44.2	2,692	45.1
Sex	Female	1,187	55.8	3,282	54.9
Race and ethnicity	Mexican American	168	7.9	428	7.2
Race and ethnicity	Other Hispanic	194	9.1	619	10.4
Race and ethnicity	Non-Hispanic White	1,171	55.1	3,503	58.6
Race and ethnicity	Non-Hispanic Black	329	15.5	710	11.9
Race and ethnicity	Non-Hispanic Asian	111	5.2	329	5.5
Race and ethnicity	Other or multiracial	154	7.2	385	6.4
Education	<9th grade	99	4.7	273	4.6
Education	9th-11th grade	218	10.2	442	7.4
Education	College graduate or above	545	25.6	2,074	34.7

Diabetes analysis inclusion profiles

Profile denominators: 2,127 excluded and 5,974 included. Category percentages use the corresponding profile denominator.

Characteristic	Category	Excluded count	Excluded (%)	Included count	Included (%)
Education	High school or equivalent qualification	537	25.2	1,201	20.1
Education	Missing at age ≥20	15	0.7	2	0.0
Education	Not eligible: age 18-19	109	5.1	231	3.9
Education	Some college or associate degree	604	28.4	1,751	29.3
Family income-to-poverty ratio	<1.30	418	19.7	1,156	19.4
Family income-to-poverty ratio	≥3.50	535	25.2	2,091	35.0
Family income-to-poverty ratio	1.30-<3.50	570	26.8	1,962	32.8
Family income-to-poverty ratio	Missing	604	28.4	765	12.8
Disability status	No disability	1,707	80.3	4,862	81.4
Disability status	Disability	420	19.7	1,112	18.6

Hypertension analysis inclusion profiles

Profile denominators: 1,929 excluded and 6,172 included. Category percentages use the corresponding profile denominator.

Characteristic	Category	Excluded count	Excluded (%)	Included count	Included (%)
Age (years)	18-29	362	18.8	877	14.2
Age (years)	30-44	452	23.4	1,299	21.0
Age (years)	45-64	524	27.2	2,022	32.8
Age (years)	65 and older	591	30.6	1,974	32.0
Sex	Male	842	43.6	2,790	45.2
Sex	Female	1,087	56.4	3,382	54.8
Race and ethnicity	Mexican American	162	8.4	434	7.0
Race and ethnicity	Other Hispanic	185	9.6	628	10.2
Race and ethnicity	Non-Hispanic White	1,068	55.4	3,606	58.4
Race and ethnicity	Non-Hispanic Black	281	14.6	758	12.3
Race and ethnicity	Non-Hispanic Asian	96	5.0	344	5.6
Race and ethnicity	Other or multiracial	137	7.1	402	6.5
Education	<9th grade	86	4.5	286	4.6
Education	9th-11th grade	194	10.1	466	7.6
Education	College graduate or above	509	26.4	2,110	34.2
Education	High school or equivalent qualification	482	25.0	1,256	20.3
Education	Missing at age ≥20	13	0.7	4	0.1
Education	Not eligible: age 18-19	83	4.3	257	4.2

Hypertension analysis inclusion profiles

Profile denominators: 1,929 excluded and 6,172 included. Category percentages use the corresponding profile denominator.

Characteristic	Category	Excluded count	Excluded (%)	Included count	Included (%)
Education	Some college or associate degree	562	29.1	1,793	29.1
Family income-to-poverty ratio	<1.30	360	18.7	1,214	19.7
Family income-to-poverty ratio	≥3.50	496	25.7	2,130	34.5
Family income-to-poverty ratio	1.30-<3.50	506	26.2	2,026	32.8
Family income-to-poverty ratio	Missing	567	29.4	802	13.0
Disability status	No disability	1,570	81.4	4,999	81.0
Disability status	Disability	359	18.6	1,173	19.0

Disability prevalence by demographic subgroup

Characteristic	Subgroup	N	Disability n	Disability (%)	95% lower (%)	95% upper (%)	Missing / not asked	Precision note
Age (years)	18-29	1,239	153	12.5	10.6	14.6	0	
Age (years)	30-44	1,751	188	9.8	7.6	12.4	0	
Age (years)	45-64	2,546	521	18.7	16.2	21.5	0	

Disability prevalence by demographic subgroup

Characteristic	Subgroup	N	Disability n	Disability (%)	95% lower (%)	95% upper (%)	Missing / not asked	Precision note
Age (years)	65 and older	2,565	670	25.0	22.5	27.7	0	
Sex	Male	3,632	599	13.8	11.7	16.2	0	
Sex	Female	4,469	933	18.9	16.6	21.4	0	
Race and ethnicity	Mexican American	596	85	12.0	8.3	17.1	0	
Race and ethnicity	Other Hispanic	813	169	17.9	15.3	21.0	0	
Race and ethnicity	Non-Hispanic White	4,674	914	17.6	15.6	19.9	0	
Race and ethnicity	Non-Hispanic Black	1,039	180	13.5	10.4	17.3	0	
Race and ethnicity	Non-Hispanic Asian	440	45	5.7	3.1	10.3	0	
Race and ethnicity	Other or multiracial	539	139	23.9	18.5	30.3	0	
Education	<9th grade	372	134	30.6	24.1	38.1	357	
Education	9th-11th grade	660	204	28.4	24.8	32.3	357	
Education	High school or equivalent qualification	1,738	412	21.7	19.5	24.1	357	
Education	Some college or associate degree	2,355	464	17.6	15.7	19.6	357	
Education	College graduate or above	2,619	273	8.0	6.5	9.8	357	
Family income-to-poverty ratio	<1.30	1,574	454	26.1	24.0	28.3	1,369	

Disability prevalence by demographic subgroup

Characteristic	Subgroup	N	Disability n	Disability (%)	95% lower (%)	95% upper (%)	Missing / not asked	Precision note
Family income-to-poverty ratio	1.30-<3.50	2,532	539	19.6	18.0	21.4	1,369	
Family income-to-poverty ratio	≥3.50	2,626	298	9.9	8.1	12.1	1,369	

Included/excluded composition describes the observed sample using unweighted counts. Subgroup disability prevalence uses each demographic subgroup as its denominator and uses interview weights (WTINT2YR). Education applies only to adults aged ≥20.

Disability domains under both thresholds, by cohort

Threshold labels: Some or worse means some difficulty or worse. A lot or cannot means a lot of difficulty or cannot do at all.

Domain	Cohort	Difficulty threshold	N	Positive n	Prevalence (%)	95% lower (%)	95% upper (%)	Weight
Seeing	Interview	Some or worse	8,101	3,183	37.0	34.4	39.7	Interview
Seeing	Interview	A lot or cannot	8,101	366	3.7	3.1	4.4	Interview
Hearing	Interview	Some or worse	8,101	1,967	21.9	19.9	24.2	Interview
Hearing	Interview	A lot or cannot	8,101	239	2.6	2.2	3.0	Interview
Walking or climbing steps	Interview	Some or worse	8,101	2,642	27.2	24.9	29.7	Interview
Walking or climbing steps	Interview	A lot or cannot	8,101	779	7.3	6.4	8.4	Interview

Disability domains under both thresholds, by cohort

Threshold labels: Some or worse means some difficulty or worse. A lot or cannot means a lot of difficulty or cannot do at all.

Domain	Cohort	Difficulty threshold	N	Positive n	Prevalence (%)	95% lower (%)	95% upper (%)	Weight
Communicating	Interview	Some or worse	8,101	1,215	13.5	11.9	15.3	Interview
Communicating	Interview	A lot or cannot	8,101	150	1.5	1.2	1.9	Interview
Remembering or concentrating	Interview	Some or worse	8,101	3,763	44.6	42.3	46.9	Interview
Remembering or concentrating	Interview	A lot or cannot	8,101	538	6.5	5.6	7.5	Interview
Self-care	Interview	Some or worse	8,101	710	7.4	6.4	8.6	Interview
Self-care	Interview	A lot or cannot	8,101	122	1.2	1.0	1.5	Interview
Any of six domains	Interview	Some or worse	8,101	5,830	68.7	66.3	70.9	Interview
Any of six domains	Interview	A lot or cannot	8,101	1,532	16.4	14.5	18.6	Interview
Seeing	Diabetes analysis	Some or worse	5,974	2,394	37.4	34.3	40.6	Phlebotomy
Seeing	Diabetes analysis	A lot or cannot	5,974	255	3.4	2.6	4.5	Phlebotomy
Hearing	Diabetes analysis	Some or worse	5,974	1,508	22.7	20.3	25.3	Phlebotomy
Hearing	Diabetes analysis	A lot or cannot	5,974	170	2.6	2.2	3.1	Phlebotomy
Walking or climbing steps	Diabetes analysis	Some or worse	5,974	1,945	27.4	25.0	30.0	Phlebotomy

Disability domains under both thresholds, by cohort

Threshold labels: Some or worse means some difficulty or worse. A lot or cannot means a lot of difficulty or cannot do at all.

Domain	Cohort	Difficulty threshold	N	Positive n	Prevalence (%)	95% lower (%)	95% upper (%)	Weight
Walking or climbing steps	Diabetes analysis	A lot or cannot	5,974	537	7.0	6.0	8.2	Phlebotomy
Communicating	Diabetes analysis	Some or worse	5,974	881	13.6	11.9	15.5	Phlebotomy
Communicating	Diabetes analysis	A lot or cannot	5,974	95	1.3	0.9	1.8	Phlebotomy
Remembering or concentrating	Diabetes analysis	Some or worse	5,974	2,854	46.0	43.8	48.2	Phlebotomy
Remembering or concentrating	Diabetes analysis	A lot or cannot	5,974	399	6.7	5.7	7.9	Phlebotomy
Self-care	Diabetes analysis	Some or worse	5,974	481	7.1	5.9	8.6	Phlebotomy
Self-care	Diabetes analysis	A lot or cannot	5,974	56	0.8	0.6	1.2	Phlebotomy
Any of six domains	Diabetes analysis	Some or worse	5,974	4,372	69.6	67.2	72.0	Phlebotomy
Any of six domains	Diabetes analysis	A lot or cannot	5,974	1,112	16.6	14.1	19.4	Phlebotomy
Seeing	Hypertension analysis	Some or worse	6,172	2,493	37.7	34.8	40.6	Examination
Seeing	Hypertension analysis	A lot or cannot	6,172	273	3.5	2.8	4.6	Examination
Hearing	Hypertension analysis	Some or worse	6,172	1,563	22.9	20.6	25.4	Examination

Disability domains under both thresholds, by cohort

Threshold labels: Some or worse means some difficulty or worse. A lot or cannot means a lot of difficulty or cannot do at all.

Domain	Cohort	Difficulty threshold	N	Positive n	Prevalence (%)	95% lower (%)	95% upper (%)	Weight
Hearing	Hypertension analysis	A lot or cannot	6,172	179	2.6	2.2	3.1	Examination
Walking or climbing steps	Hypertension analysis	Some or worse	6,172	2,024	27.5	25.0	30.2	Examination
Walking or climbing steps	Hypertension analysis	A lot or cannot	6,172	573	7.2	6.1	8.4	Examination
Communicating	Hypertension analysis	Some or worse	6,172	922	13.7	12.0	15.6	Examination
Communicating	Hypertension analysis	A lot or cannot	6,172	103	1.4	1.0	1.9	Examination
Remembering or concentrating	Hypertension analysis	Some or worse	6,172	2,948	46.1	44.0	48.2	Examination
Remembering or concentrating	Hypertension analysis	A lot or cannot	6,172	424	6.8	5.8	8.0	Examination
Self-care	Hypertension analysis	Some or worse	6,172	510	7.2	6.0	8.6	Examination
Self-care	Hypertension analysis	A lot or cannot	6,172	61	0.9	0.6	1.2	Examination
Any of six domains	Hypertension analysis	Some or worse	6,172	4,523	69.8	67.4	72.1	Examination

Disability domains under both thresholds, by cohort

Threshold labels: Some or worse means some difficulty or worse. A lot or cannot means a lot of difficulty or cannot do at all.

Domain	Cohort	Difficulty threshold	N	Positive n	Prevalence (%)	95% lower (%)	95% upper (%)	Weight
Any of six domains	Hypertension analysis	A lot or cannot	6,172	1,173	16.8	14.4	19.6	Examination

Six domains and any-domain prevalence are shown at both thresholds. The main disability definition uses a lot of difficulty or cannot do at all. Cohort-specific percentages use their own sampling weights.
